# Experimental analysis of the thermal management and internal quantum efficiency of terahertz quantum cascade laser harmonic frequency combs

**DOI:** 10.1515/nanoph-2025-0207

**Published:** 2025-07-28

**Authors:** M. Alejandro Justo Guerrero, Elisa Riccardi, Lianhe Li, Mark Rosamond, A. Giles Davies, Edmund H. Linfield, Miriam S. Vitiello

**Affiliations:** NEST, CNR – Istituto Nanoscienze and Scuola Normale Superiore, Piazza San Silvestro 12, 56127, Pisa, Italy; School of Electronic and Electrical Engineering, University of Leeds, Leeds LS2 9JT, UK

**Keywords:** quantum cascade laser, frequency combs, photoluminescence, thermal and electronic properties

## Abstract

Quantum cascade laser (QCL) harmonic state frequency combs (HFCs) have recently attracted considerable interest for applications ranging from the generation of tones of high spectral purity, high data rate wireless communication networks, radiofrequency arbitrary waveform synthesis, and for fundamental light-matter investigations in quantum physics. However, a detailed knowledge of the nature of the electronic and thermal energy distribution in these devices is of paramount importance for the refinement of their thermal management and quantum efficiency, which are key to the widespread adoption of QCL HFC technology in a new generation of integrated optical quantum platforms. Here, we perform a comparative study of the thermal and electronic properties of Fabry–Perot and micro-ring HFC QCLs, operating in the terahertz frequency range, using micro-probe band-to-band photoluminescence. By monitoring the lattice temperature and the electron cooling above the threshold for stimulated emission, we extract the device thermal resistances and the internal quantum efficiencies, highlighting the key role of the resonator architecture.

## 1 Introduction

Optical frequency combs (FCs) operating at terahertz (THz) frequencies have emerged as a transformative tool for spectroscopy [1], [2], quantum communications [3], far-field and near-field imaging [4], materials science, metrology [5], [6], [7] and for fundamental investigations in physics, chemistry and engineering. Characterized by a set of coherent and uniformly spaced spectral lines, FCs offer unparalleled capabilities in the precise generation and manipulation of THz radiation [8], [9], [10], including spontaneous non-linear intracavity phenomena in miniaturized semiconductor heterostructure lasers [5], [6], [8]. The recent experimental demonstration of spontaneous generation of high harmonic order frequency combs (HFCs) in THz frequency quantum cascade lasers (QCLs) [10] has extended the range of applications of QCL FCs to future wireless THz communication networks [3], promising intracavity generation of powerful terahertz carrier signals, whose frequency can be designed by engineering the device cavity [11], [12], [13] or through suitable facet coatings [14].

In the mid-infrared, QCL HFCs have been engineered as Fabry–Perot (FP) or ring-shaped cavities with localized defects [12], [13], [15], [16]. Each geometry possesses its respective advantages and disadvantages; for example, FP-QCLs have a simpler design, wide tunability, and a cheaper manufacturing cost compared to ring QCLs [17], [18]. Unlike a FP resonator that supports FP longitudinal modes, a ring micro-resonator supports the formation of whispering gallery modes (WGM), modes guided by total internal reflection along the outer perimeter of the ring. In this latter case, confinement is higher and losses are low, causing, in the laser, a decreased threshold current density and a reduced heat load, as well as quality (*Q)* factors larger than that of FP-QCLs [19], [20]. THz QCL FC or HFC, fabricated in ridge-type waveguides, usually produce a generally frequency-modulated (FM) output with an almost constant intensity and a linear frequency chirp [21], due to the large nonlinear susceptibility *χ* ^3^ of the core semiconductor heterostructure. In FP QCL, broadband emission is caused by spatial hole burning (SHB) [22] that has also negative side effects, such as linewidth broadening [23], mode-hopping, and wavelength chirping, that severely prevent the exploitation of the whole gain bandwidth of the laser. In a ring, governed by WGM, FC operation can be either SHB-driven, as in presence of localized defects, or, alternatively FC dissipative Kerr solitons can be produced in defect-free ring QCLs. The latter are particularly suitable for on-chip photonic integration, making this technology appealing in the far-infrared, where scattering losses become negligible and the confinement is larger than in the visible, near- or mid-infrared ranges. Indeed, miniaturized photonic quantum integrated platforms, in the THz frequency range, with accurate thermal management, are ideal for quantum sensing and quantum communications applications [1], [2], [3], [4], [5], [6], [7]. But, it is critical to have an accurate knowledge of the thermal and electronic management in THz frequency QCL FCs if the intriguing prospect of their widespread integration into on-chip photonic platforms is to be realized.

A direct comparison between the thermal and electronic performance of FP and ring shaped QCL HFCs has not been performed to date in the THz frequency range, owing to the fundamentally different nature of the modes that support laser action and the resulting differences in the intracavity four wave mixing process [24] responsible for the phase locking mechanisms of the multi-modes arising from spatial hole burning [25]. Here, we perform a sophisticated experimental analysis of the thermal and electronic properties of Fabry Perot and micro-ring QCL HFCs operating at THz frequencies. By comparing the lattice and electronic temperatures of structures having an identical active region, we extract the device thermal resistances and their internal quantum efficiencies [26], shedding light on the essential differences between the two types of HFC source.

**Figure 1:**
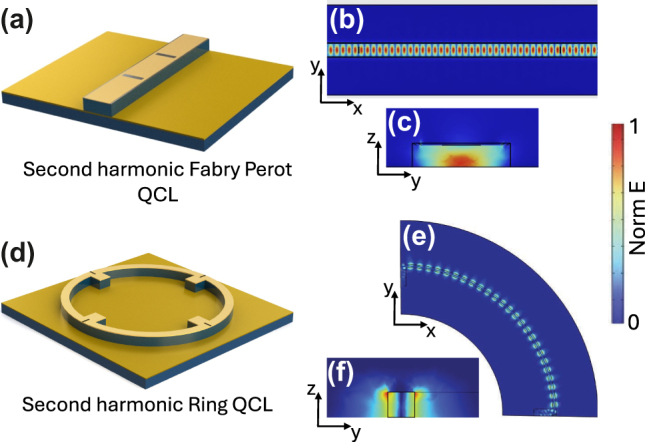
Device schematics. (a, d) Three-dimensional device schematics of samples A and B, respectively. (b, c, e, f) Top and cross-section views of the normalized electric field distribution inside sample A (b and c respectively), and sample B (e and f respectively), taken from three-dimensional (3D) finite element modelling (FEM) simulations.

In our work, we use a method that alleviates the influence of device fabrication on the specific operational FC state, and allows setting of the number of lasing modes. In the present case, the spatial dependence of the intermode beat intensity along the cavity is defined by defect engineering the top laser surface. Specifically, our FP HFCs have a regularly distributed sequence of defects patterned on the top laser surface (Figure 1a) [12], [16] the same approach being used for micro-ring HFCs [15] have a cavity architecture in which a set of four equidistant pads is distributed at π/2 intervals (Figure 1d), resulting in stable second harmonic generation across the device’s entire dynamic range.

## 2 Results and discussions

For a reliable comparison, we use the same active region (AR) design for both the FP (sample A) and micro-ring (sample B) QCLs; this consists of a 17 µm thick GaAs/AlGaAs heterostructure, grown by molecular beam epitaxy (MBE) on a semi-insulating GaAs substrate. The QCLs are identical to those described in Refs. [27], [28] design based on three frequency-shifted active region modules exploiting LO-phonon-assisted interminiband transitions [27].

The devices were fabricated into metal–metal waveguided structures. Fabrication started with an Au–Au thermocompression wafer bonding of the AR to a highly doped GaAs substrate. The laser bars/rings were then defined using a combination of optical lithography, metal deposition, and dry etching. The FP laser bars were 76 µm wide and 2.5 mm long, while the micro-rings had a 1.5 mm external diameter and an internal width of 15 µm (4.7 mm perimeter). Individual QCLs were indium soldered onto copper mounts and wire bonded for their transport and optical characterization (see Supplementary information). Figure 1b, c, e, and f show the top/cross-section views of the simulated spatial distribution of the normalized electric fields (see Supporting information) in samples A and B, respectively. As expected, the electric field distribution shows that while FP QCL supports TM_00_ modes, the ring QCL supports WGMs, an important difference between these two classes of device.

To compare the thermal and electronic properties of the HFCs, we performed microprobe band-to-band photoluminescence (PL) spectroscopy, which have been successfully employed, previously, for the study of the electronic, thermal, and vibrational properties of mid-IR and THz QCLs [29], [30], [31], operating in continuous wave (CW). For both samples, data were acquired both below and well above the laser threshold.

PL spectra were measured using a confocal Raman spectrometer (Horiba, XploraPlus) equipped with a 638 nm laser in a backscattering configuration, with the visible red light focused through a microscope objective lens (100×) onto the center of the QCL facet along the 17 µm thick growth axis. Under these experimental conditions, the beam had a circular spot of ∼10 µm diameter, and was fully focused on the central active region of the heterogeneous stack [28], which emits at a central frequency of 3 THz, according to the design reported in ref. [28]. We kept the laser-induced electron heating below ∼5 K, by using an incident optical power density of ∼10 kW/cm^2^, thus leaving the electronic distribution practically unperturbed by the incident radiation. In QCL ARs, the electron energy distribution is determined by the balance between the input power from tunneling injection and the different energy relaxation channels, which include inter- and intrasubband *e*-*e*, *e*-phonon, and e-impurity scattering, as well as interface-roughness scattering [30], which, at THz frequencies, is playing a non-negligible role.

**Figure 2:**
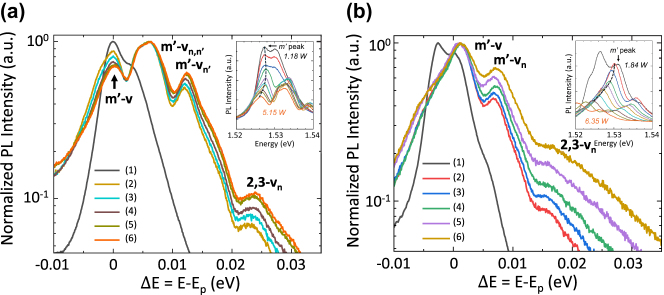
Photoluminescence spectra measured in (a) sample A and (b) sample B, at different values of current density, plotted as a function of the energy difference from the main PL bands (E_p_). For sample A: (1) J ≈ 3 A/cm^2^, (2) J ≈ 241 A/cm^2^, (3) J ≈ 300 A/cm^2^, (4) J ≈ 378 A/cm^2^, (5) J ≈ 462 A/cm^2^, and (6) J ≈ 534 A/cm^2^. For sample B: (1) J ≈ 3 A/cm^2^, (2) J ≈ 259 A/cm^2^, (3) J ≈ 300 A/cm^2^, (4) J ≈ 385 A/cm^2^, (5) J ≈ 471 A/cm^2^, and (6) J ≈ 546 A/cm^2^. The heat sink temperature was kept at 20 K. Inset: Data plotted as a function of the energy. The evolution of the main peak, attributed to band-to band transitions involving the miniband m^’^ is marked with a dashed line.

The insets of Figure 2a and b shows a set of representative PL spectra measured in samples A and B, respectively, for different values of the dissipated electrical power (P), at a fixed heat sink temperature of 20 K. In both cases, at zero bias, a double peak band associated with radiative transitions involving two closely spaced (3 meV) subbands in the injector miniband [27] is observed (peak *m’*), with the PL peaks spanning about 20 meV. Under bias, when electrons are tunnel injected into the upper laser level, additional features appear on the high energy tail of the PL band associated with the injector miniband and arise from band-to-band recombination between conduction and valence subbands. Furthermore, the intensity of the main PL bands (E_P_) decreases owing to tunnel injection from the injection miniband state and the upper laser level. As a result of the combined effect of Joule heating and Stark shift induced by the applied electric field, *m* ^*’*^ shifts with the dissipated electrical power. The estimated electric field effect is ΔE = 0.27 meV (0.11 meV) for sample A(B).

To aid comparison, each spectrum is also plotted as a function of the energy difference ΔE with respect to the corresponding E_P_ value, and the PL intensity normalized (Figure 2a and b; main figures). Above threshold (*J* = 250 A/cm^2^), all PL peaks redshift as a consequence of the heating of the electrons, whilst the gradient of the high energy slopes of the spectra increases as a function of applied voltage; these characteristics can be used as thermometric properties to determine the local lattice (*T*_*L*_) and the electronic temperatures (*T*_*e*_), respectively [30], [31], [32].

The cavity architecture has a significant influence on the shape of the PL peaks. In the ring cavity laser (Figure 2b), there are three main peaks: the first corresponds to interband transitions involving two subbands at the bottom of the miniband *m’* and a valence subband *v*. The second peak, separated by a distance of 8 meV, is ascribed to a conduction-to valence subbands transition still involving the 17.7 meV wide miniband *m’* (Ref [27]), but a different valence subband *v* ^*’*^. The final peak, labelled as *2,3*, is 17–20 meV away from the main peak and it is ascribed to interband transitions involving the upper laser level (2,3 in ref [27]).

For the Fabry Perot laser (Figure 2a), we observe more structured PL spectra. The lowest energy peak corresponds again corresponds to interband transitions between the bottom of the miniband *m’* and a valence subband, *v.* There is, then, a broader (∼2.5 meV width) convolution of three peaks, 6 meV higher in energy, and a peak separated by ∼14 meV, which are also ascribed to interband transitions involving *m* ^’^. The final, weaker, peak at ∼25 meV is attributed to transitions starting from the upper laser states *2,3*. Importantly, since the intensity of the PL peaks depends on the populations of the conduction and valence subbands, and on the overlap integral of the envelope functions, the latter is at an identical bias point for the two devices. The larger intensity of the high energy peak in the Fabry–Perot cavity indicates a greater population of the upper laser state. Indeed, the analysis of the PL lineshape is based on the following expression:

$$I_{PL}\left(E\right)\propto \overset{5}{\underset{j=1}{\sum }}\overset{4}{\underset{k=1}{\sum }}A_{jk}E_{jk}^{4}{\left|\left\langle \Psi_{j},|,\Psi_{k}\right\rangle \right|}^{2}L\left(E\right)$$

where *A*_*jk*_ = *n*_*j*_ ⋅ *p*_*k*_, *n*_*j*_ and *p*_*k*_ are the populations of the conduction and valence subbands. The term is the overlap integral of the envelope functions. The lineshape function *L(E)* is obtained joining a Lorentzian with a phenomenological broadening Γ/2, on the low energy side, and an exponential decay $\propto \exp\left|-E/k_{B}T_{e}^{j}\right|$ on the high energy side.

In order to extract *T*_*L*_, we compared the redshift of the main PL peak as a function of the dissipated electrical power (*P*) with the position (in energy) of the same peak, at zero bias, as a function of the heat sink temperature (calibration curve), which was varied with an accuracy of ±0.5 K.

**Figure 3:**
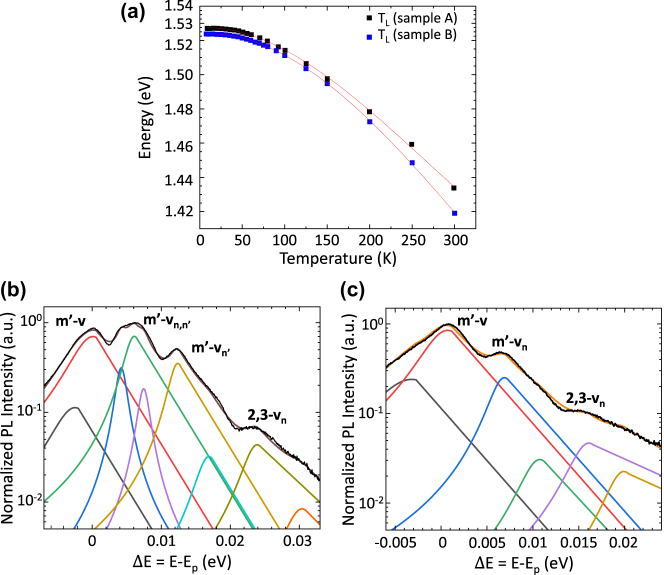
Calibration curve and lineshape analysis. (a) Thermal calibration curves measured by tracking the redshift of the main peak in the photoluminescence spectra from sample A (black squares) and sample B (blue squares) as a function of the heat sink temperature, at zero bias. The red curves are 3^rd^-order polynomial fits to each data set. (b–c): Photoluminescence spectra at *J* ≈ 362 A/cm^2^ measured from samples A and B, respectively. The black curves represent experimental data, whereas the color curves show the fitting curves (half lorentzian, half exponential). The brown curve in b, and the orange curve in c, depict the cumulative function of the multiple peak fitting. The coefficient of determination (R^2^) obtained from the fittings (b and c) is 0.976 and 0.989, respectively.

Figure 3a shows the resulting thermal calibration curves of samples A and B (black and blue squares, respectively). Both curves were corrected to compensate for the field effect, ΔE = 0.27 meV (0.11 meV) in the FP-HFC (ring-HFC), occurring at low bias [33] – this typically plays a role up to the threshold for alignment and leads to an applied electric field induced shift of the confinement energies in the conduction and valence bands. The slight difference between the calibration curves can be attributed to minor differences in the devices thermal management ascribed to the indium soldering of the QCLs onto the copper chips, which provide the thermal contact to the heat sink and affects the local device temperature.

The electronic temperature, *T*_*e*_, was calculated using a deconvolution process on the PL spectra. Prototypical examples are shown in Figure 3b and c for samples A and B, respectively, at the same current density. The line shape function, utilized for the multiple peak fitting of the PL spectra, is obtained by merging a Lorentzian function with a phenomenological broadening (Γ/2) on the low energy side, with an exponential decay function $\propto \exp\left[-\Delta E/k_{B}T_{e}\right]$ on the high energy side [34], where Δ*E* is the energy, and *k*_*B*_ is the Boltzmann constant. The experimental data was then fitted by leaving *T*_*e*_, Γ/2, and the peak intensities as fitting parameters.

The two peaks at ΔE = 4.2 meV and ΔE = 7.5 meV, shown in Figure 3b, are ascribed to the heavy-hole (HH) and light-hole (LH) excitons arising from the ground-state miniband. This assignment is supported by the following facts: (i) the energy separation (HH-LH ∼4 meV) is coincident with the HH-LH splitting of excitons detected in 13–15 nm wide GaAs quantum wells [35]; and (ii) the line shape is Lorentzian [36], [37] with a small linewidth of about 1.5 meV, as shown in Figure 3b. The observation of excitons associated with ground-state subbands in PL spectra is a well-studied subject. Experiments show that the threshold concentration at which the collapse of excitons due to the charge induced screening of the Coulomb potential takes place is sample dependent, and has a value of n_c_∼10^10^ cm^−2^ in quantum structures grown under the same experimental conditions as the investigated devices [38]. In our structure, excitons associated with the levels outside *m’* cannot be observed because they exist in doped quantum wells for which the electron sheet density is > n_c_. Conversely, the wave function of the bottom levels of *m’* mainly exist in a spatial region (four rightmost quantum wells) [26] characterized by a negligible static electronic density. It is notable that excitonic transitions are not seen in the case of the ring HFC (Figure 3c), even though the active region is the same. This is likely to be a result of a lower intensity, that is hidden by the more intense main PL peaks, possibly as a result of local variations in growth parameters across different portions of the MBE wafer.

**Figure 4:**
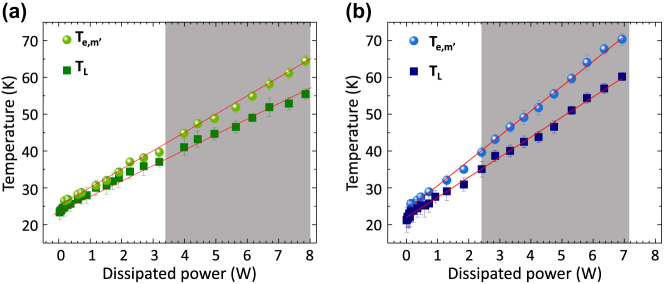
Lattice temperatures (*T*_*L*_) and ground state subband electronic temperatures (*T*_*e,m’*_) in the active region of (a) sample A and (b) sample B, measured as a function of the dissipated power (*P*) with the heat sink temperature kept at 20 *±* 0.5 K. The red lines are linear fits to the data. The shaded areas show the lasing region.

Figure 4a and b displays the variation of the lattice and electronic temperatures of miniband *m*’, for samples A and B, respectively, as a function of the dissipated electrical power. At a heat sink temperature of 20 K, the electronic temperatures of all subbands belonging to *m’* (Figure 3b and c) are nearly equal, and both increase linearly with dissipated electrical power with slopes of $R_{e}^{\left(A\right)}=5.76\;K/W$ and $R_{e}^{\left(B\right)}=7.56\;K/W$ for samples A and B respectively, slightly larger than the corresponding thermal resistance variations, $R_{L}^{\left(A\right)}=4.36\;K/W$ and $R_{L}^{\left(B\right)}=5.45\;K/W$ .

To better compare the thermal properties of the two types of HFCs investigated, we extracted the normalized thermal resistance $R^{*}=R\times \left(S/d\right)$ , where *S* and *d* represent the device area and active region thickness, respectively. We obtained $R_{L}^{*}\left(A\right)=4.9\;Kcm/W$ and $R_{L}^{*}\left(B\right)=3.0\;Kcm/W$ . Sample B exhibits a lower normalized thermal resistance than sample A, indicating a higher capacity to reduce the heat load of the active region during CW operation, and hence better thermal management. Owing to the formation of whispering gallery modes (WGM), confinement is indeed higher in the ring and, consequently, losses are lower. In lasers, this leads to a decreased threshold current density, that we have also observed in the present case (sample A: *J*_*th*_ ≈ 300.49 Acm^−2^, sample B: *J*_*th*_ ≈ 287.63 Acm^−2^). The lower threshold current, in turn, reduces the required electrical pumping and leads to a decreased heat load of the active medium, in agreement with our experimental observations.

The experimental measurement of the electronic and lattice temperatures were then used as a self-calibrated method to evaluate the internal efficiency (*η*_int_) [26] of our QCLs. By adopting a simple rate equation model we can assume that: i) the average excess energy of the active region (injector) is the difference between the energy carried by the incoming and the outgoing electrons, which leave the active region (injector) with an average energy $kT_{AR}^{e}$ ( $kT_{\mathrm{inj}}^{e}$ ); and, ii) far from the energy resonance the electron-electron (*e-e*) energy relaxation terms coupling the injector and active region subsystems can be neglected with respect to the *e*-lattice ones. Accordingly, the following energy balance equations can be written for the active region (AR):

(1) $$\begin{array}{ll} \frac{\text{d}E_{AR}}{dt} & =\frac{J}{q}\left(kT_{\mathrm{inj}}^{e}-kT_{AR}^{e}+\Delta E_{2,3-m^{\prime }}+\Delta E_{m^{\prime }}\right)\\ & \;-\frac{kn_{AR}}{\tau_{eL}}\left(T_{AR}^{e}-T_{L}\right)-g\Delta nSh\upsilon \end{array}$$

where $\Delta {\text{E}}_{m^{\prime }}$ is the miniband width, $\Delta E_{2,3-m^{\prime }}$ is the energy separation between the upper state and the miniband m’ (lower laser level), *S* is the photon flux, and *g* = *α*_tot_/Δ*n* the gain cross section, related to the total losses, *α*_*tot*_, in the laser cavity. In the present active region design, the energy difference $kT_{\mathrm{inj}}^{e}-kT_{AR}^{e}$ << $\Delta E_{2,3-m^{\prime }}+\Delta E_{m^{\prime }}$ and can thus be neglected in equation (1).

Taking the derivative of equations (1) in steady state below (S = 0) and above the laser threshold gives:

(2) $$\frac{d\left(T_{AR}^{e}-T_{L}\right)}{dJ}\approx \frac{\tau_{eL}}{qn_{AR}k}\left(\Delta E_{2,3-m^{\prime }}+\Delta E_{m^{\prime }}\right)$$

(3) $$\frac{d\left(T_{AR}^{e}-T_{L}\right)}{dJ}\approx \frac{\tau_{eL}}{qn_{AR}k}\left(h\upsilon +\Delta E_{m^{\prime }}-h\upsilon \eta_{\mathrm{int}}\right)$$

From equation (3) we then get:

(4) $$\eta_{\mathrm{int}}\approx 1+\frac{\Delta E_{m^{\prime }}}{h\upsilon }-\frac{qn_{AR}k}{h\upsilon \tau_{eL}}\frac{d\left(T_{AR}^{e}-T_{L}\right)}{dJ}$$

where *hν* is the emitted photon energy (that we here assume to be equal to the central frequency of the optical bandwidth of the probed AR module of the QCL FC, i.e. 3 THz), $\Delta {\text{E}}_{m^{\prime }}$ represents the quasi-miniband width (≈18 meV) in the conduction band, τ_*eL*_ is the energy loss lifetime between the system and the lattice, *n*_*AR*_ is the subband population, *q* represents the electron charge, and $T_{AR}^{e}-T_{L}$ is the difference between the electronic temperature of the upper laser level, and the lattice temperature [32].

**Figure 5:**
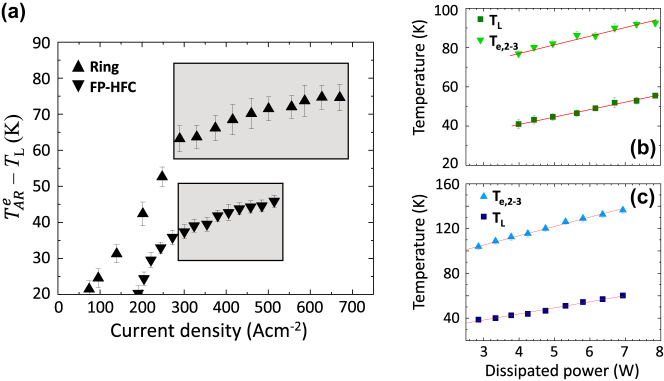
Subband electronic and lattice temperatures. (a) Difference between the electronic temperatures of the upper state and the lattice temperature as a function of current density in samples A and B (down and up triangles, respectively). (b, c) Electronic temperatures (upper state) and lattice temperature measured as a function of the dissipated power in samples A (b) and B (c), respectively.

Figure 5a shows the difference $T_{AR}^{e}-T_{L}$ evaluated for samples A and B, plotted as a function of the current density, where $T_{AR}^{e}$ has been assumed equal for both samples A and B to the electronic temperature of the upper laser levels 2,3. The shadow areas represent the lasing regions. The slope reduction observed in the $T_{AR}^{e}-T_{L}$ trend for both types of QCL HFCs, as a function of the current density, comes from the abrupt change in the slope of the electronic temperatures $T_{AR}^{e}$ measured above the lasing threshold of samples A and B (Figure 5b and c). This behavior is consistent with the decrease of the electron heating rate when photon emission extracts part of the power supplied to the laser, and consequently, the electrons are cooling down more efficiently, in agreement with previous reports [32]. The slope $\frac{d\left(T_{AR}^{e}-T_{L}\right)}{dJ}$ above lasing threshold, extracted from the data in Figure 5a, for sample A (0.038 K/Acm^−2^) and B (0.033 K/Acm^−2^) show that for sample A the laser cooling is less effective.

The derivative of $\left(T_{AR}^{e}-T_{L}\right)$ as a function of the current density (*J*) was evaluated from the data in Figure 5a, and used in equation (3) to extract *η*_int_ [26] taking advantage of the experimental observation that the ratio $\frac{\tau_{eL}}{qk_{B}n_{AR}}$ is approximately constant below the lasing threshold (*S* = 0) (Supplementary information). The latter was extracted inserting the experimental data of Figure 5b and c into the steady state solution of equation (1):

(5) $$\frac{qnk_{B}}{\tau_{eL}}=\left(\Delta E_{2,3-m^{\prime }}+\Delta E_{m^{\prime }}\right){\left[\frac{d\left(T_{AR}^{e}-T_{L}\right)}{dJ}\right]}^{-1}$$

The internal efficiencies calculated for samples A and B are (59 ± 5)% and (71 ± 8)%, respectively. Being *n*_int_ = *n*_*p*_ ⋅ *n*_*tr*_, where *n*_*tr*_ is the transition efficiency and *n*_*p*_ the total pumping efficiency [39], we can extract *n*_*p*_ = 1 − *J*_*leak*_/*J*_*th*_, where *J*_*leak*_ and *J*_*th*_ are the carrier-leakage and threshold-current density, respectively. *n*_*tr*_ can be indeed extracted from the formula $n_{2,3}/\left(n_{2,3}+n_{m}^{\prime }\right)$ and for the present active region design [28], [40] it results approximately equal to 94 %. Being *n*_*tr*_ an inherent property of the active region, this reflects in different *n*_*p*_ values between sample A and B. In the present case, we extracted *n*_*p*_ ∼ 63 % and *n*_*p*_ ∼ 75 % for sample A and B respectively, meaning that the ring QCL displays a more efficient pumping efficiency, as an effect of the different device architecture. The internal efficiency is also directly related with other fundamental physical parameters, central in the theory of lasers sources, such as the differential external efficiency $\eta_{d}=\frac{\alpha_{m}}{\alpha_{\mathrm{tot}}}\eta_{\mathrm{int}}$ and the slope efficiency $\frac{dP_{0}}{dI}=\eta_{\mathrm{int}}\frac{\alpha_{m}}{\alpha_{\mathrm{tot}}}\frac{Nh\nu }{e}$ , where *η*_int_ and *η*_*d*_ refer to a single QCL period, *N* is the number of periods, *α*_*m*_ are the mirror losses, and *α*_*tot*_ = *α*_*w*_ + *α*_*m*_, where *α*_*w*_ are the waveguide losses. The use of whispering-gallery modes lowers the mirror losses and increases the cavity quality factor; however, in our case, the presence of the contact pads in the internal perimeter of the ring QCL (sample B) leads to calculated values of *α*_*m*_ = 2 cm^−1^, and *α*_*w*_ = 10 cm^−1^. In contrast, in the 2.5 mm long double metal cavity of sample A, the calculated mirror and waveguide losses are *α*_*m*_ = 1.9 cm^−1^ and *α*_*w*_ = 23 cm^−1^, respectively; these values agree with previous reported values [41], [42]. In the present case, at T_H_ = 20 K, we found *η*_*d*_ = 4.4 ± 0.4 % and *η*_*d*_ = 12 ± 1 % for samples A and B, respectively. Corresponding, slope efficiencies values were: $\frac{dP_{0}}{dI}=\left(35\pm 2\right)\frac{mW}{A}$ for sample A, and $\frac{\text{d}P_{0}}{\text{d}I}=\left(83\pm 6\right)\frac{mW}{A}$ for sample B. The latter values are a factor ∼3.5 and ∼7.5 larger than those obtained by conventional optical measurements [43], [44] (see Supporting information), which are inherently limited by the small collection efficiencies of the optical set-ups and the optical beam divergence that, especially in a double-metal waveguides, heavily affect a careful evaluation.

## Supplementary Material

Supplementary Material Details
