# Supplementary material for: Experimental analysis of the thermal management and internal quantum efficiency of terahertz quantum cascade laser harmonic frequency combs
Source: Nanophotonics. 2025 Jul 28;14(23):4249–57. doi: 10.1515/nanoph-2025-0207 (PMC12617731; doi:10.1515/nanoph-2025-0207)
Supplement: Supplementary file 1 — Supplementary Material Details [file j_nanoph-2025-0207_suppl_001.docx]

**Supplementary Information**

**Experimental analysis of the thermal management and internal quantum efficiency of terahertz quantum cascade laser harmonic frequency combs**

M. Alejandro Justo Guerrero, ^1^ Elisa Riccardi, ^1^ Lianhe Li,^2^ Mark Rosamond,^2^ A. Giles Davies,^2^ Edmund H. Linfield,^2^ and Miriam S. Vitiello^1*^

*^1^ NEST, CNR - Istituto Nanoscienze and Scuola Normale Superiore, Piazza San Silvestro 12, 56127, Pisa, Italy*

*^2^School of Electronic and Electrical Engineering, University of Leeds, Leeds LS2 9JT, UK*

1. **Finite element method simulations**

The frequency-domain finite element method simulations were performed using a commercial software package (Comsol Multiphysics 6.1), with the wave optics module in the eigenfrequency mode. The 3D simulations of the harmonic frequency comb (HFC) ring quantum cascade laser (QCL), sample B, were performed assuming a 1.5 mm external diameter and a 15 µm width. The ring cavity includes four 60 µm x 100 µm pads, radially located every $\pi/2$, with one rectangular slit each (3 µm x 15 µm), centered with respect to the pad and aligned to the external ring diameter (Fig. S1). The simulation of the HFC Fabry-Pérot (FP) QCL, sample A, consisted of a ridge cavity of 80 µm width and 2.5 mm length. The FP QCL also includes two rectangular slits (2 μm × 42 μm) and two, 3 μm wide, nickel side absorbers aligned along the edges of the top surface of the cavity (Fig.S1).

The active region of both samples A and B has a thickness of 17 µm. To allow for the free propagation of the electric field away from the cavity surfaces, both devices were enclosed in a larger volume, as shown in Fig. S1. The active region refractive index was set to *n*_AR_ = 3.6, with the real and imaginary parts of the refractive indexes of the nickel defects and side absorbers set to *n*_Ni_ = 30 and *k*_Ni_ = 50, respectively. The surroundings of the cavity volume were defined using a refractive index *n*_Air_ = 1. As the simulations are performed in the terahertz (THz) frequency range, the top and bottom boundaries of the cavity are considered perfect electric conductors to emulate the gold interfaces. The nickel side absorbers and defects boundaries are hence defined using the transition boundary condition, with the nickel layer thickness being 5 nm. All the external boundaries were assumed to be scattering boundaries to emulate infinite large surroundings.

**Figure S1**: 3D models of (a) sample A and (b) sample B as defined in the Comsol simulations. The insets show an expanded view around the Ni slits on the top contacts of both samples A and B.

1. **Current-voltage (I-V) and light-current (L-I) characteristics**

The current-voltage (I-V) and light-current (L-I) characteristics of the devices presented in the main text are reported in Figs. S2a and S2b. Compared to the HFC FP-QCL, the HFC ring-QCL show a consistent threshold current density (*J*_th_), a ≈10% increase of the slope efficiency and a ≈ 7% increase of the maximum wall plug efficiency (WPE) (see Table I).


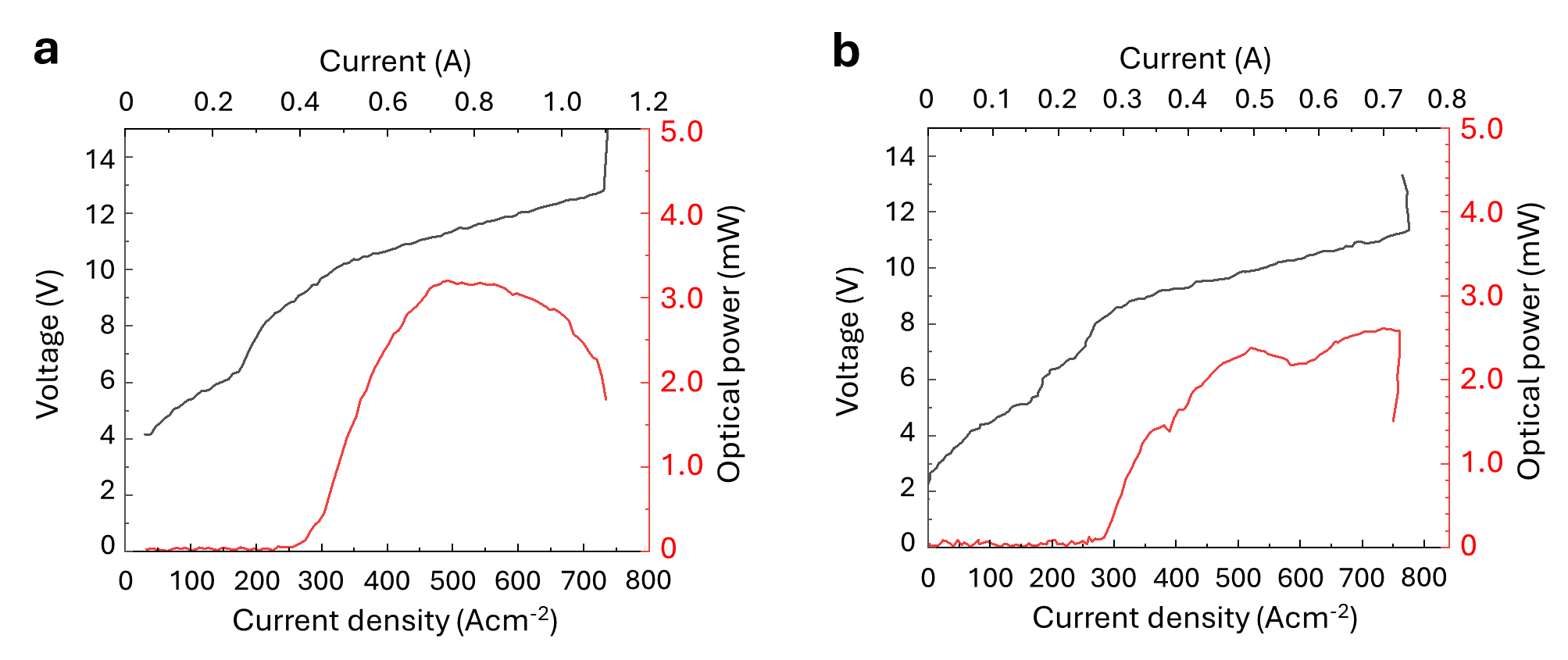


**Figure S2**. Current-voltage (I-V) and light-current (L-I) characteristics of: (a) the FP-HFC QCL (sample A) and (b) the ring QCL (sample B).

|  | **J_th_ (A/cm^2^)** | **Slope Eff. (mW/A)** | **Max WP Eff. (%)** | **Peak power (mW)** |
| --- | --- | --- | --- | --- |
| **Sample A** | 300.49 | 10 | 0.037 | 3.2 |
| **Sample B** | 287.63 | 11 | 0.040 | 2.6 |

**Table I.** Figures of merit of the devices described in the main text.

**3. Spectral behavior characteristics**

Figure S3 shows the spectral response of the lasers studied in the main text, the FP (S3a) and Ring QCLs (S3b). In the spectra from both devices, it is possible to observe the same harmonic comb behavior with a frequency spacing between the modes of ≈30 GHz.


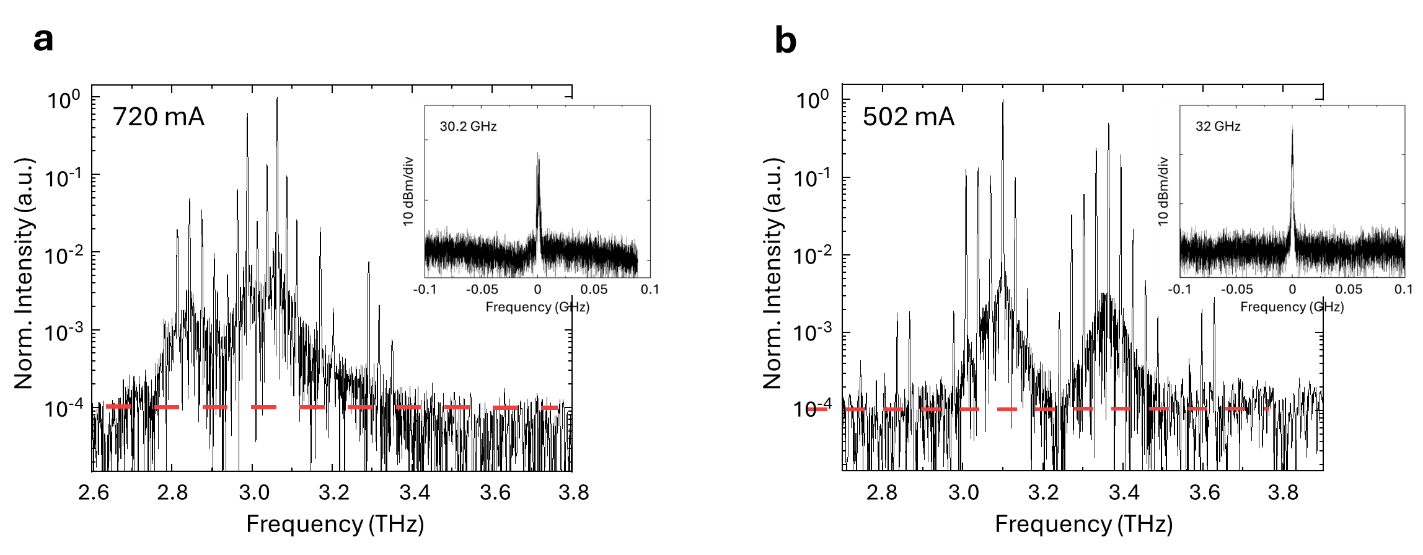


**Figure S3.** Fourier transform infrared emission spectra of the (a) FP-HFC QCL and (b) ring QCL. Insets: intermode beatnote signal extracted from the bias line with a bias-tee and recorded with an RF spectrum analyzer (Rohde and Schwarz FSW; RBW: 500 Hz, video bandwidth (VBW): 500 Hz, sweep time (SWT): 20 ms, RMS acquisition mode). All measurements are performed in CW, while driving the QCLs at the currents marked on the graphs at a fixed heat sink temperature of 15 K.
